# Identification of ferroptosis-related molecular clusters and genes for diabetic osteoporosis based on the machine learning

**DOI:** 10.3389/fendo.2023.1189513

**Published:** 2023-08-14

**Authors:** Xingkai Wang, Lei Meng, Juewei Zhang, Zitong Zhao, Linxuan Zou, Zhuqiang Jia, Xin Han, Lin Zhao, Mingzhi Song, Junwei Zong, Shouyu Wang, Xueling Qu, Ming Lu

**Affiliations:** ^1^ Department of Trauma and Tissue Repair Surgery, Dalian Municipal Central Hospital, Dalian, China; ^2^ Department of Orthopaedic Surgery, The First Affiliated Hospital of Dalian Medical University, Dalian, China; ^3^ Department of Surgery, The First Affiliated Hospital of Nanhua Medical University, Hengyang, China; ^4^ Health Inspection and Quarantine, College of Medical Laboratory, Dalian Medical University, Dalian, China; ^5^ International Department, Beijing No.80 High School, Beijing, China; ^6^ Department of Surgery, The First Affiliated Hospital of Dalian Medical University, Dalian, China; ^7^ Department of Surgery, Naqu People's Hospital, Tibet, China; ^8^ Department of Orthopaedic Surgery, The Second Affiliated Hospital of Dalian Medical University, Dalian, China; ^9^ Department of Quality Management, Dalian Municipal Central Hospital, Dalian, China; ^10^ Changjianglu Pelvic Floor Repair Center, Dalian Women and Children’s Medical Group, Dalian, China

**Keywords:** diabetic osteoporosis, ferroptosis, molecular clusters, machine learning, prediction model

## Abstract

**Background:**

Diabetic osteoporosis exhibits heterogeneity at the molecular level. Ferroptosis, a controlled form of cell death brought on by a buildup of lipid peroxidation, contributes to the onset and development of several illnesses. The aim was to explore the molecular subtypes associated with ferroptosis in diabetic osteoporosis at the molecular level and to further elucidate the potential molecular mechanisms.

**Methods:**

Integrating the CTD, GeneCards, FerrDb databases, and the microarray data of GSE35958, we identified ferroptosis-related genes (FRGs) associated with diabetic osteoporosis. We applied unsupervised cluster analysis to divide the 42 osteoporosis samples from the GSE56814 microarray data into different subclusters based on FRGs. Subsequently, FRGs associated with two ferroptosis subclusters were obtained by combining database genes, module-related genes of WGCNA, and differentially expressed genes (DEGs). Eventually, the key genes from FRGs associated with diabetic osteoporosis were identified using the least absolute shrinkage and selection operator (LASSO), Boruta, support vector machine recursive feature elimination (SVM ­ RFE), and extreme gradient boosting (XGBoost) machine learning algorithms. Based on ROC curves of external datasets (GSE56815), the model’s efficiency was examined.

**Results:**

We identified 15 differentially expressed FRGs associated with diabetic osteoporosis. In osteoporosis, two distinct molecular clusters related to ferroptosis were found. The expression results and GSVA analysis indicated that 15 FRGs exhibited significantly different biological functions and pathway activities in the two ferroptosis subclusters. Therefore, we further identified 17 FRGs associated with diabetic osteoporosis between the two subclusters. The results of the comprehensive analysis of 17 FRGs demonstrated that these genes were heterogeneous and had a specific interaction between the two subclusters. Ultimately, the prediction model had a strong foundation and excellent AUC values (0.84 for LASSO, 0.84 for SVM ­ RFE, 0.82 for Boruta, and 0.81 for XGBoost). IDH1 is a common gene to all four algorithms thus being identified as a key gene with a high AUC value (AUC = 0.698).

**Conclusions:**

As a ferroptosis regulator, IDH1 is able to distinguish between distinct molecular subtypes of diabetic osteoporosis, which may offer fresh perspectives on the pathogenesis of the disease’s clinical symptoms and prognostic heterogeneity.

## 1 Introduction

According to the World Health Organization, diabetes prevalence rates have been rising over the past few decades. Currently, more than 463 million people worldwide have diabetes, and by 2040, that figure is projected to double (1). Not only is diabetes dangerous in its own right, but its numerous complications also have a severe impact on patients. Diabetic osteoporosis, as a chronic complication of the skeletal system, can lead to major pain and skeletal deformities, as well as high levels of disability and mortality, making the treatment and rehabilitation of diabetic patients more difficult, not only affecting the quality of life but also increasing the financial burden (2, 3). Unfortunately, less attention has been paid to osteoporosis than to the complications traditionally associated with diabetes such as macrovascular disease and microvascular disease. Consequently, further exploration of the pathogenesis and prevention of diabetic osteoporosis is warranted.

In contrast to apoptosis, necroptosis, and autophagy, ferroptosis is a unique kind of iron-dependent programmed cell death that is characterized by an excessive buildup of reactive oxygen species and lipid peroxidation (4). Ferroptosis has been shown to be associated with the pathophysiological mechanisms underlying several illnesses and contribute to the onset and progression of such diseases. A growing amount of research is now confirming that ferroptosis may be a valuable research direction for the prevention and treatment of osteoporosis (5–7). Nevertheless, the molecular subtypes and processes relating to ferroptosis in diabetic osteoporosis need to be elucidated. Thus, comprehending the connection between FRGs and the development of diabetes osteoporosis and locating molecular clusters based on FRGs helps to understand the molecular heterogeneity of diabetic osteoporosis.

**Figure 1:**
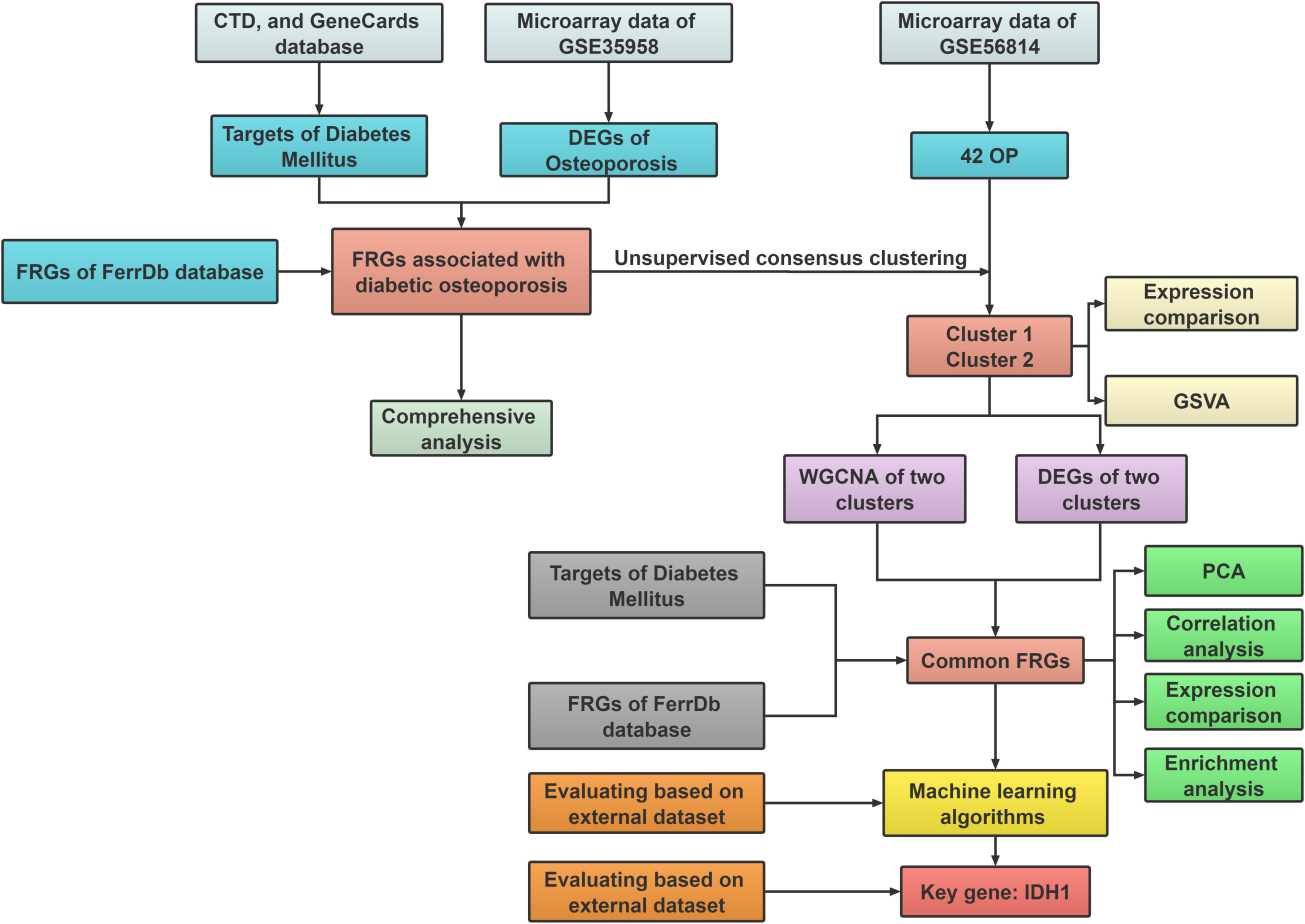
The flow chart of the study design and analysis: DEGs analysis and subcluster identification; Comprehensive analysis; Machine learning and validation.

In the current work, we thoroughly identified and in-depth investigated FRGs that differed in expression between healthy people and patients with diabetic osteoporosis. 42 osteoporosis patients were separated into two ferroptosis-associated subclusters based on 15 distinct ferroptosis gene expression profiles linked to diabetic osteoporosis, and the molecular interactions between the two clusters were further investigated. In addition, utilizing multiple machine learning methods, a robust prediction model was established and validated by employing an external dataset. The receiver operating characteristic (ROC) curve results demonstrated that FRGs could be a prospective predictor of diabetic osteoporosis subtypes. **Figure 1** depicts the research flowchart for this study.

## 2 Materials and methods

### 2.1 Data source and processing

Through applying the R package of GEOquery, three microarray datasets (GSE35958, GSE56814, and GSE56815) related to osteoporosis were downloaded from the Gene Expression Omnibus (GEO) database (http://www.ncbi.nlm.nih.gov/geo/) (8). The detailed sample information for the three datasets is presented in **Table 1**. Taking adj. P-Value < 0.05 and |log2fold change (FC)| ≥ 1 as the cutoff value, the GSE35958 dataset was selected to be used for differential gene expression analysis, which is based on the limma package in the R software (9). We then screen diabetes-related genes from two disease databases, including Comparative Toxicogenomics Database (CTD) (http://ctdbase.org/) (10), and GeneCards (https://www.genecards.org/) (11). In addition, ferroptosis-related genes (FRGs) were obtained from the FerrDb database (http://www.zhounan.org/ferrdb/) (12). Finally, 15 FRGs associated with diabetic osteoporosis were obtained as the differentially expressed genes (DEGs) between control and osteoporosis samples, and a comprehensive analysis was performed.

**Table 1:** The detailed sample information for the three datasets.

Dataset	Platform	Count	Osteoporosis	Control
GSE35958	GPL570 [HG-U133_Plus_2] Affymetrix Human Genome U133 Plus 2.0 Array	9	5	4
GSE56814	GPL5175 [HuEx-1_0-st] Affymetrix Human Exon 1.0 ST Array [transcript (gene) version]	82	42	40
GSE56815	GPL96 [HG-U133A] Affymetrix Human Genome U133A Array	80	40	40

### 2.2 Unsupervised clustering of FRGs associated with diabetic osteoporosis

To further divide the osteoporosis into different subclusters based on the expression level of the 15 FRGs associated with diabetic osteoporosis, an unsupervised cluster analysis was performed to distinguish the 42 osteoporosis samples in the GSE56814 dataset into distinct clusters by applying the R package of ConsensusClusterPlus (13). The ideal cluster number was confirmed using the CDF (cumulative distribution function) and the area under the CDF curve.

### 2.3 Expression and gene set variation analysis

We observed the expression of 15 FRGs associated with diabetic osteoporosis in different subclusters by drawing the heatmap and violin plot. We used GSVA, an unsupervised, non-parametric algorithm (14), to assess the corresponding biological characteristics and pathway activity of different ferroptosis subclusters of osteoporosis. The “c2.cp.kegg.v7.4.symbols.gmt” and “c5.go.v2022.1.Hs.symbols.gmt” gene sets were utilized for the GSVA enrichment analysis, and biological functions and pathways were judged to be substantially enriched when the adjusted p-value was less than 0.05.

### 2.4 Weighted gene co-expression network analysis

We applied the R package of WGCNA to construct a co-expression network of all genes in 42 osteoporosis samples (15). Firstly, the gene expression matrix is loaded in the R software to check for missing values and identify outliers. Secondly, we construct a scale-free network to select a soft threshold value, which is considered to be the parameter cutoff value for the construction of the adjacency matrix. A network relationship is usually defined by an adjacency matrix. Thirdly, we transformed the adjacency matrix into a topology matrix allowing similarities between genes to be represented at the expression and network topology levels. To discover gene co-expression modules, block module function, and module division analyses were lastly carried out. The association between each module and osteoporosis was determined, and the most pertinent modules were sorted based on the Pearson correlation analysis results. The genes in these modules were thought to be osteoporosis-related module genes.

### 2.5 Identifying DEGs of ferroptosis subclusters

The R package of limma in Bioconductor was used to identify DEGs by comparing the expression values between different ferroptosis subclusters. The criteria were adj. P-Value < 0.05 and |log2fold change (FC)| ≥ 1, and genes fulfilling this condition were determined to be DEGs. The asymptotic volcano map and heatmap displaying the DEGs were created using the ggplot2 and pheatmap package, respectively.

### 2.6 Comprehensive analysis of FRGs associated with diabetic osteoporosis of ferroptosis subclusters

By integrating diabetes-related genes from the CTD and GeneCards databases, FRGs from the FerrDb database, DEGs, and osteoporosis-related module genes between ferroptosis subclusters, altogether 17 FRGs associated with diabetic osteoporosis of ferroptosis subclusters were obtained. We then evaluate the efficiency of 17 genes using principal component analysis (PCA). The ggcorrplot package and the ggplot2 package were applied for the correlation analysis of 17 genes and expression analysis in different subclusters. Enrichment analysis of gene ontology (GO) and Kyoto Encyclopedia of Genes and Genomes (KEGG) pathways enrichment analysis were executed using Metascape (https://metascape.org/gp/index.html), which is a public database of gene annotation and analysis resources (16).

### 2.7 Robust prediction model created with a variety of machine learning techniques

We applied the R packages “glmnet,” “caret,” “Boruta” and “XGBoost” to build a machine learning model (17). The entire dataset of 17 genes was subjected to the least absolute shrinkage and selection operator (LASSO), Boruta, Support Vector Machine Recursive Feature Elimination (SVM ­ RFE), and extreme gradient boosting (XGBoost) analyses to identify key genes that belong to four predictive models. The 40 osteoporosis samples in GSE56815 were similarly divided into two different subclusters by 15 FRGs associated with diabetic osteoporosis and used as an external dataset to validate the efficiency of the four predictive models and key genes by the ROC curves.

## 3 Results

### 3.1 Identification of FRGs specifically expressed in diabetic osteoporosis

**Figure 2:**
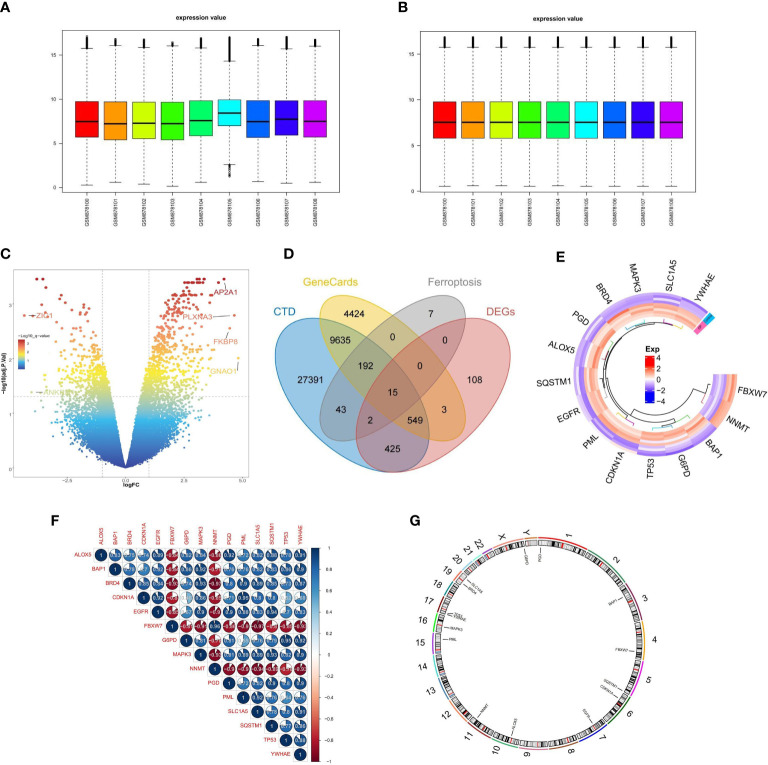
Differential FRGs screening in diabetic osteoporosis. **(A, B)** Normalization of gene expression data in samples, before **(A)** and after **(B)** normalization. **(C)** Volcano plot of DEGs between osteoporosis and controls. **(D)** Venn plot showing 15 FRGs associated with diabetic osteoporosis by intersecting the DEGs with diabetes-related genes and ferroptosis-related genes. **(E)** Representative heatmap of 15 FRGs associated with diabetic osteoporosis between osteoporosis and controls. **(F)** Representative correlation plot of 15 FRGs associated with diabetic osteoporosis. Blue represents positive correlation, and red represents negative correlation. The area of the pie chart represents the specific value of correlation coefficients. **(G)** The location of 15 FRGs associated with diabetic osteoporosis on chromosomes.

The expression profile data of 4 control samples and 5 osteoporosis samples in GSE35958 were normalized using the “limma” package (**Figures 2A, B**). Based on the adj. P-Value < 0.05 and |log2fold change (FC)| ≥ 1, a total of 1102 DEGs, including 677 up-regulated and 425 down-regulated genes, were identified by differential analysis (**Figure 2C**). We obtained 3,8253 and 1,4818 diabetes-related genes from the CTD database and the GeneCards database, respectively. The 259 ferroptosis-related genes (FRGs) were obtained from the FerrDb database. We overlapped the DEGs, diabetes-related genes, and ferroptosis-related genes, 15 overlapped genes were obtained, namely FRGs associated with diabetic osteoporosis, which was shown by the Venn diagram (**Figure 2D**). The details of these genes were shown in **Supplementary Table S1**. We can observe that the 15 FRGs associated with diabetic osteoporosis are significantly differentially expressed between the osteoporotic samples and the control samples by drawing a heat map (**Figure 2E**). To clarify the relationship between the 15 FRGs, Spearman correlation analysis was employed (**Figure 2F**). In addition, the localization of the 15 FRGs on the chromosome is shown in the loop graph (**Figure 2G**).

### 3.2 Identification of ferroptosis subclusters in osteoporosis

**Figure 3:**
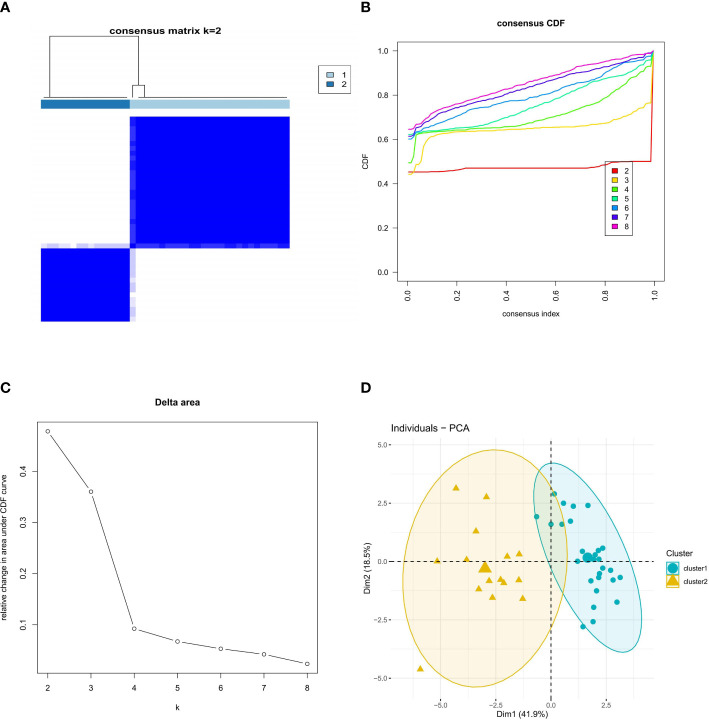
Identification of molecular clusters based on FRGs in osteoporosis. **(A)** Consensus clustering matrix of FRGs when k = 2. **(B)** Cumulative distribution function (CDF) plot when k value ranges from 2 to 8. **(C)** Relative change in the area under the CDF curve for k values from 2 to 8. **(D)** PCA of FRGs in the osteoporosis samples (cluster 1 is marked in blue and cluster 2 in yellow).

We explored the ferroptosis subclusters in osteoporosis using unsupervised clustering to analyze the expression of 15 FRGs associated with diabetic osteoporosis in 42 osteoporotic samples. The number of subtypes is most stable when k = 2 of the consensus matrix, representing the two well-defined clusters (**Figure 3A**). As shown in **Figure 3B**, the CDF curve for k = 2 has minimal fluctuations in the consistency index range of 0-1.0. The CDF diagram showed the relative change in area for variable values of k (**Figure 3C**). Principal component analysis (PCA) further supported the finding that the two clusters differed considerably (**Figure 3D**).

### 3.3 Differences between ferroptosis subclusters

**Figure 4:**
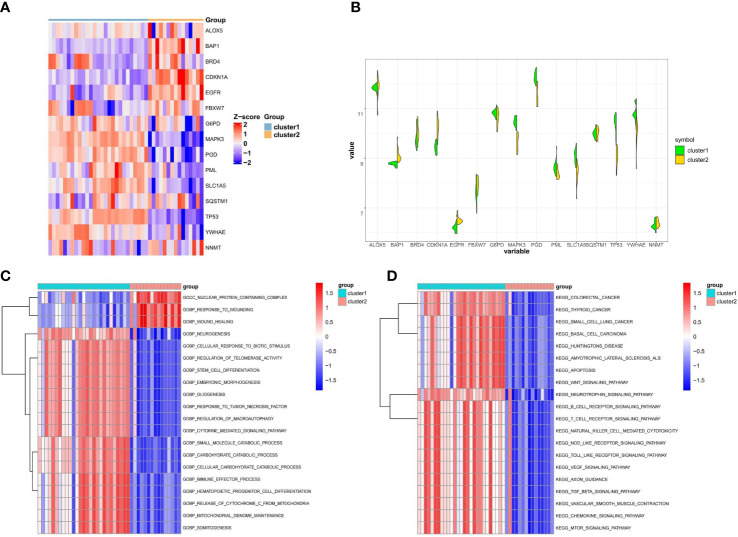
Difference analysis between the two ferroptosis clusters. **(A)** Representative heatmap of 15 FRGs between the two ferroptosis clusters. **(B)** Violin plot of 15 FRGs expression between the two ferroptosis clusters. **(C, D)** GSVA enrichment analysis in different ferroptosis clusters showing biological functions **(C)** and significantly activated pathways **(D)**.

To better understand the distinctions between the two ferroptosis subclusters, we analyzed the expression differences and variations in the pathway and biological activity of the 15 FRGs in the two subclusters. The expression of 15 FRGs between the two subclusters was apparently distinguished from that in the control and osteoporotic samples (**Figure 4A**). Cluster1 exhibited higher expression levels of FBXW7, G6PD, MAPK3, PML PGD, SLC1A5, SQSTM1, TP53, and YWHAE, while Cluster1 exhibited higher expression levels of ALOX5, BAP1, BRD4, CDKN1A, EGFR and NNMT (**Figure 4B**). Biological function results from GSVA analysis showed that nuclear protein containing complex, response to wounding, and wound healing were downregulated in Cluster1, while neurogenesis, cellular response to biotic stimulus, and regulation of telomerase activity were upregulated in Cluster2 (**Figure 4C**). In addition, the enrichment pathway of Cluster1 is mainly upregulated, such as in colorectal cancer, thyroid cancer, and small cell lung cancer, while Cluster2 is mainly associated with the downregulation pathway, like basal cell carcinoma, huntingtons disease, and amyotrophic lateral sclerosis als (**Figure 4D**). The details of the GSVA analysis were shown in **Supplementary Table S2**. These findings showed that there are notable differences between ferroptosis clusters of osteoporosis patients in terms of the expression, enriched pathways, and biological roles of 15 FRGs. For various subclusters of ferroptosis, specific therapeutic approaches are needed.

### 3.4 Differential genes analysis between ferroptosis clusters

**Figure 5:**
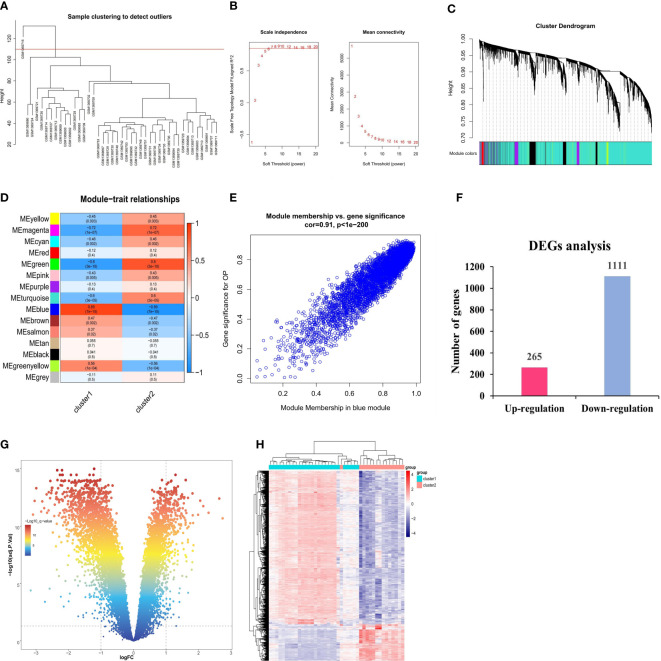
Identification of DEGs between ferroptosis subclusters. **(A)** Sample clustering dendrogram. **(B)** Analysis of scale-free fitting index and average connectivity for various soft-threshold powers (β). **(C)** Identification of co-expression gene modules. The branches of the dendrogram cluster into 15 modules and each one was labeled in a unique color. **(D)** Representative module-trait heatmap was established based on the eigenvalues values of the modules. **(E)** Representative scatter plot showing the correlation between the blue module and module-related. **(F)** Quantitative results of the number of upregulated and downregulated DEGs. **(G)** Volcano plot of DEGs between two ferroptosis subclusters. **(H)** Heatmap of DEGs between two ferroptosis subclusters.

The possible modules with the strongest connections to the ferroptosis subclusters were constructed by the WGCNA algorithm, based on the gene expression profiles. As shown in **Figure 5A**, the sample with the serial number GSM1369716 was excluded as an outlier. The ideal soft threshold for maintaining a network with scale-free topology was determined to be 6 (R2 = 0.85) (**Figure 5B**). Based on correlation clustering, the 15 signature modules were categorized and given various color labels (**Figure 5C**). The blue module (4,591 genes) had the strongest connection with Cluster1 (R = -0.89) and Cluster2 (R = -0.89) among these modules (**Figure 5D**). We observed a significant correlation between the blue module and the module-related genes (cor = 0.91) (**Figure 5E**). Subsequently, we identified the DEGs of the ferroptosis subclusters using adj. P-Value < 0.05 and |log2fold change (FC)| ≥ 1 as the cutoff value. 1,376 DEGs in total were found; 265 of them showed up-regulation, whereas 1,111 showed down-regulation (**Figures 5F–H**).

### 3.5 Comprehensive analysis of FRGs associated with diabetic osteoporosis of ferroptosis subclusters

**Figure 6:**
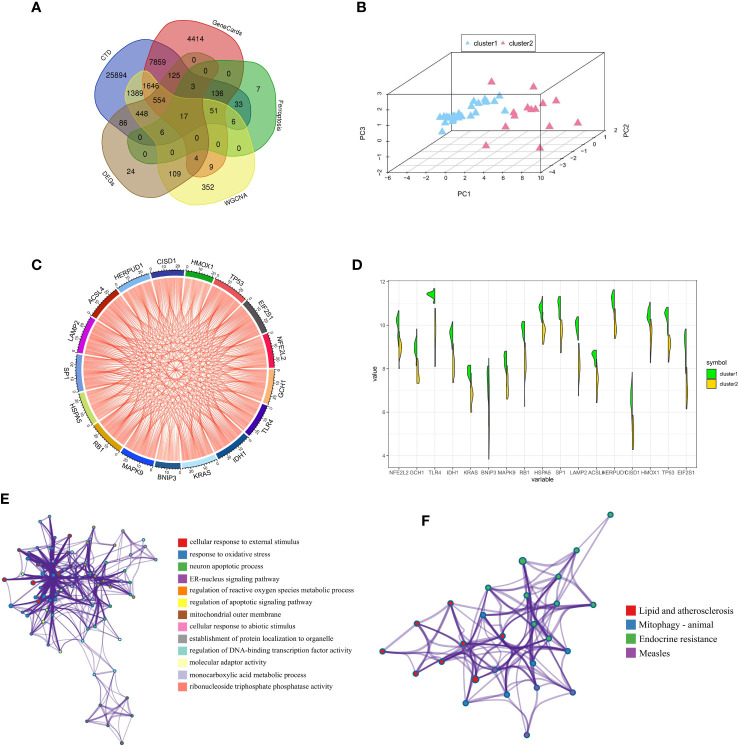
Comprehensive analysis of FRGs between ferroptosis subclusters. **(A)** Venn plot showing 17 FRGs associated with diabetic osteoporosis by intersecting the DEGs and module-related genes with diabetes-related genes and ferroptosis-related genes. **(B)** PCA of 17 FRGs showing good differentiation power between ferroptosis subclusters. **(C)** Representative gene relationship network diagram of 17 FRGs associated with diabetic osteoporosis. **(D)** Violin plot of 17 FRGs expression between the two ferroptosis clusters. **(E)** The GO enrichment analyses results. Nodes represent description. **(F)** The KEGG enrichment analyses results. Nodes represent description.

We obtained a total of 17 FRGs associated with diabetic osteoporosis of ferroptosis subclusters, by combining genes from the database and dataset (**Figure 6A**). The details of genes were shown in **Supplementary Table S3**. The PCA results revealed 17 FRGs associated with diabetic osteoporosis which effectively distinguish between the two ferroptosis subclusters (**Figure 6B**). In addition, the relationship network diagram of the 17 FRGs associated with diabetic osteoporosis showed a significant positive correlation with each other, contributing to a comprehensive analysis of the interrelationship among the genes (**Figure 6C**). Meanwhile, all genes except BNIP3 were highly expressed in cluster1, as shown in **Figure 6D**. GO and KEGG enrichment analyses were carried out to further investigate the probable biological function and pathway activity of the 17 FRGs relevant to diabetic osteoporosis. The significant results from GO enrichment analysis revealed that 17 FRGs were primarily associated with cellular response to external stimulus, response to oxidative stress, and neuron apoptotic process (**Figure 6E**). Moreover, 17 FRGs were mainly involved in various classical signaling pathways based on the KEGG enrichment analyses, including Lipid and atherosclerosis, Mitophagy ­ animal, and Endocrine resistance (**Figure 6F**). **Supplementary Table S4** displayed the findings of the GO and KEGG analyses in detail.

### 3.6 Construction of prediction model and identification of key gene

**Figure 7:**
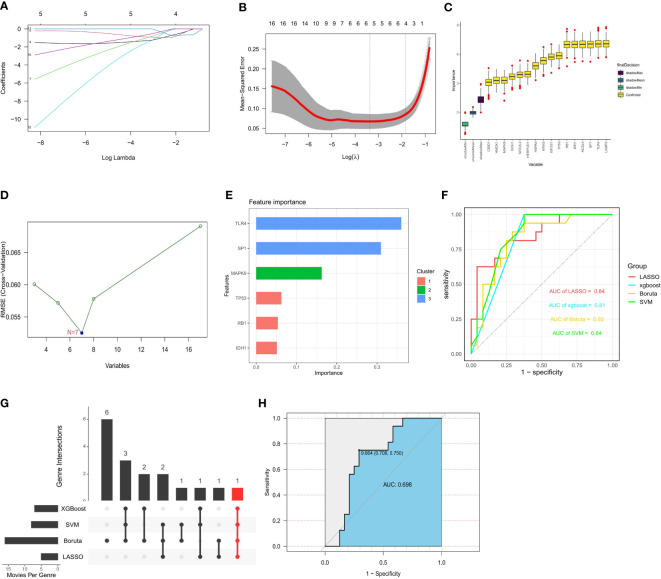
Construction of prediction model and identification of key gene. **(A, B)** 4 FRGs obtained using the LASSO algorithm based on the minimum lambda. **(C)** 7 FRGs obtained using the SVM algorithm. **(D)** 16 FRGs obtained using the Boruta algorithm. **(E)** 6 FRGs obtained using the XGBoost algorithm. **(F)** Applying external dataset to validate four predictive models. **(G)** The common gene belonging to all four algorithms. **(H)** Applying external dataset to validate the key gene.

Based on the whole dataset, we used four established machine learning methods (LASSO, SVM ­ RFE, Boruta, and XGBoost) to find important genes from 17 FRGs associated with diabetic osteoporosis. These algorithms yielded 4, 7, 16, and 6 genes, respectively (**Figures 7A–E**). We then verified the efficiency of the four machine learning algorithms by ROC curves utilizing GSE56815 as an external dataset. All four algorithms had high area under curve (AUC) values that were more than 0.8 and we considered the results of the prediction models to be reliable (**Figure 7F**). IDH1 is a common gene belonging to all four algorithms (**Figure 7G**). The details of genes were shown in **Supplementary Table S5**. Considering the accuracy of the identified genes, we plotted ROC curves with the external dataset GSE56815, which showed a high predictive efficiency (**Figure 7H**). Ultimately, we identified IDH1 from 17 FRGs as a prospective indicator of diabetic osteoporosis subtypes through four highly efficient prediction models.

## 4 Discussion

Diabetic osteoporosis, a chronic complication of diabetes in the skeletal system with a high risk of fracture, is not only a medical problem but also a critical social problem, making early detection and timely intervention extremely significant (18). The role of FRGs in the underlying pathogenesis of diabetic osteoporosis has attracted strong research interest from scholars both nationally and internationally. In addition, studies have confirmed evidence of ferroptosis in the bone tissue of diabetic osteoporotic rats, and inhibitors of ferroptosis may improve osteoporosis symptoms (19). However, the molecular mechanisms of FRGs in different molecular subtypes of diabetic osteoporosis were not widely reported. The molecular heterogeneity of diabetic osteoporosis is made more understandable by the identification of molecular clusters based on FRGs.

In the current study, we synthesized database data and gene expression profiling data for the analysis of FRGs associated with diabetic osteoporosis. In total, we identified 15 FRGs that can distinguish diabetic osteoporosis from normal samples, suggesting that ferroptosis may exert an overarching role in the pathological development of diabetic osteoporosis. Subsequently, correlation and expression analysis between ferroptosis regulators elucidated the complex relationship between diabetic osteoporosis and normal individuals. Furthermore, the expression of 15 FRGs associated with diabetic osteoporosis allowed for the classification of 42 osteoporotic samples into two different categories. Expression and enrichment analysis of the 15 FRGs exhibited significant differences between the two molecular clusters, suggesting that ferroptosis could be a prospective indicator of osteoporotic subtypes. According to GSVA enrichment analysis, cluster1 was predominantly enriched in the cellular response to biotic stimulus, regulation of telomerase activity, and stem cell differentiation. Cluster2 was prominently associated with nuclear protein containing the complex, response to wounding, and wound healing. Ferroptosis as a heterogeneous regulator of diabetic osteoporosis, we combined WGCNA and differential expression analysis to identify 17 FRGs that could effectively distinguish between the two subtypes. Ultimately, we identified IDH1 from 17 FRGs as a prospective indicator of diabetic osteoporosis subtypes, using multiple machine learning algorithms and validated by uniting external datasets.

IDH1, a gene located on chromosome 2q34, encodes the IDH1 protein consisting of 414 amino acids situated in the cytoplasm and peroxisome (20). IDH1 is a homodimer that forms an active NADP+ binding site among the structural domains (21). NADP+ dependent IDH1 catalyzes the oxidative decarboxylation of isocitrate to produce alpha-ketoglutarate, which leaves DNA and histones in a demethylated state (22). In previous studies, mutations in IDH1 were found in association with a variety of malignancies and rare cases, such as low-grade diffuse gliomas, periosteal cartilage tumours, cholangiocarcinoma, acute myeloid leukaemia, and Ollier’s disease and Maffucci’s syndrome (23). In malignancies, IDH1 mutations promote the accumulation of lipid reactive oxygen species (ROS) by reducing the protein levels of glutathione peroxidase 4 (GPX4), which subsequently leads to ferroptosis (24). This phenomenon indicates that tumour-derived IDH1 mutations sensitise cells to ferroptosis. IDH1 primarily exerts metabolic effects *in vivo*, IDH1 ectopic expression suppresses the production of brown adipocytes as a novel therapeutic target against obesity and related metabolic diseases such as type II diabetes and may represent a therapeutic target for the treatment of metabolic diseases (25). Besides metabolic functions, IDH1 also regulates gene expression through epigenetic modifications of histones. However, it has never been studied whether IDH1 affects ferroptosis and thus worsens the progression of diabetic osteoporosis. Diabetes induces fat accumulation causing atherosclerosis and narrowing of the vascular lumen, resulting in inadequate blood supply to the bone (26). With a poor vascular system, the blood supply to the bone tissue is compromised which will not work properly and structural abnormalities, such as microcracks, may occur. However, it has been demonstrated that IDH1 inhibition regulates the progression of atherosclerosis by improving macrophage viability and apoptosis, and may alleviate atherosclerosis by activating NRF2 to ameliorate ox-LDL-induced ferroptosis in macrophages (27). Furthermore, IDH1 production of NADPH in the cytoplasm reduces intracellular oxidative stress, which as a complication of diabetes can also lead to osteoporosis (28). In this study, IDH1 was identified combined with machine learning prediction models and expressed significantly different in the analysis of diabetic osteoporosis subtypes. Moreover, plotting the ROC curve with the external data set as the validation set resulted in a high AUC value (0.698). The above results sufficiently demonstrate that IDH1 has a high probability of acting as a regulator of ferroptosis in differentiating the heterogeneity of diabetic osteoporosis subtypes, which provides a strong basis for further studies.

There are certain research limitations that need to be made clear. We lacked a sufficiently large number of diabetic osteoporosis samples to assess the accuracy of ferroptosis genes in machine learning model predictions and to validate the stability of subpopulations. More prognostic information needs to be gathered in further studies to assess the prognostic value of ferroptosis in distinct subtypes of diabetic osteoporosis. Additionally, IDH1 needs further literature support and experimental validation as a regulator of iron death which distinguishes diabetic osteoporosis subtypes.

## 5 Conclusion

In conclusion, we found two clusters of ferroptosis in diabetic osteoporosis and confirmed the distinctive characteristics of each. Four distinct machine learning prediction models (LASSO, XGBoost, Boruta, and SVM) based on 17 ferroptosis genes discovered ferroptosis regulators capable of distinguishing diabetic osteoporosis subtypes. Ultimately, as a ferroptosis regulator validated by the external datasets, IDH1 has the capacity to precisely distinguish molecular subtypes of diabetic osteoporosis, which may provide novel insights into the pathophysiology of the clinical symptoms and prognostic heterogeneity in diabetic osteoporosis.

## Data availability statement

The original contributions presented in the study are included in the article/**Supplementary Material**. Further inquiries can be directed to the corresponding authors.

## Author contributions

XW, LM and JunZ collected the original data, finished the analysis and drafted the initial version. ZZ, LZ, ZJ, XH, LZ and MS helped revise the manuscript. ML and XQ offered constructive comments on experimental studies. ML, XQ, SW and JunZ provided the funding. ML designed and conceptualized the study, put forward many constructive comments for the final version and supervised the study. All authors contributed to the article and approved the submitted version.

## Supplementary material

Supplementary Table 1 The details of database genes, DEGs and intersecting genes.

Supplementary Table 2 The details of GSVA analysis.

Supplementary Table 3 The details of database genes, DEGs, module-related genes of WGCNA and intersecting genes.

Supplementary Table 4 The detailed analysis results of GO and KEGG.

Supplementary Table 5 The details of the genes obtained by four machine learning algorithms.
